# Treatment of Diphtheria, from Correspondence of the London Lancet

**Published:** 1863-12

**Authors:** 


### MISCELLANEOUS.

### TREATMENT OF DIPHTHERIA, FROM CORRESPONDENCE OF THE LONDON LANCET.

To the Editor of the Lancet:

Sir:—I have lately met with five very severe cases of diphtheria, or what was sixty years since, when I was a pupil of Dr. Harper, called angina maligna, or putrid sore-throat. I have always found it highly contagious. It affects the nervous system very early, inducing great prostration of strength and delirium; the countenance very anxious; the throat and fauces being so swollen as to threaten suffocation; the uvula, being elongated, irritates the throat, and gives a constant disposition to vomit, but without effect; the mouth and fauces appear as if boiling water had been poured down the throat, or like raw beef; the tongue covered with a thick whitish fur; a burning heat not only in those parts, but extending to the whole of the chest; and a total inability to swallow anything. Of course the first thing is to prevent suffocation, the next to get the poison out of the system, and repair the mischief it has done.

For a great many years I have found the effervescing saline with nitrate of potash can be swallowed when nothing else can. I have given it every half hour, and ice, if it can be kept in the mouth, and swallowed as it becomes dissolved. If ice cannot be got, I have ordered cold spring water, the volatile liniment to be used externally, and cold vinegar and water rags to be applied to the forehead. Where the skin is hot and the pulse high, I have ordered the body to be well sponged two or three times a day with tepid water, in which a little soda has been dissolved, and had a current of fresh air passed through the room very often, and every means used to prevent the disease spreading. These means I have always found successful, nor do I recollect to have lost one patient.

About thirty-five years since I was sent for to a family, and found one of them dead from suffocation before I got there. The others recovered. As soon as the inflammatory stage is passed, port wine and tonics may be given with advantage. I have generally found that from eight to twelve days after a person has been exposed to the infection, is the time of what may be termed the incubation; but its attack is then sudden. One of my cases was a volunteer, who was at drill on Saturday evening; but on Sunday noon, when I first saw him, he was in a very dangerous state.

I am, Sir, your obedient servant,

William Jones, M. D.

To the Editor of the Lancet:

Sir:—When a man with sixty years' experience addresses you on any subject—when, moreover, he is able to say that in the treatment of so deadly a disease as diphtheria he has always been successful, and does not recollect having lost a single case—his communication well deserves the attention of the profession. Will Dr. Jones, therefore, pardon a very much younger man than himself asking him a few questions in reference to his letter in *The Lancet* of last date?

Dr. Jones at once assumes the identity of diphtheria and the old putrid sore-throat, and says he has always found it "highly *contagious*." He further says that he has generally found the period of incubation to be "from eight to twelve days after a person has been exposed to the *infection*." Now, as it is an important point, will Dr. Jones be good enough to say whether he makes any distinction between contagion and infection? and, if so, whether he considers diphtheria infectious? If it is not asking too much, will he also give us some idea of the kind of evidence on which he bases his opinion? Accepting generally his description of the symptoms and the indications for treatment, might I ask whether he has *never* seen more than an ineffectual effort to vomit in any of his cases? And whether, above all, he has never observed the characteristic membrane from which the disease derives its modern name, and of which he makes no mention? Might I further remind him that a "total inability to swallow anything" is quite incompatible with the administration even of saline draughts, ice, or spring water, and ask if in such cases his only expedient "to prevent suffocation" has been the volatile liniment used externally.

I may add that, although I can boast of no such sucess with this hateful disease as Dr. Jones speaks of, I am quite satisfied of two things which he entirely overlooks, and these are the early administration of wine, etc., even during the "inflammatory stage," and the value of a local treatment. I have some suspicions, however, that Dr. Jones' diphtheria and mine and most other people's are somewhat different; but if he will kindly reply to the questions set down above, my suspicions may be removed, and I am sure he will have conferred a great boon on the profession as well as the public if he has found and made known a method of treatment in diphtheria which is "always successful," or as nearly so as we can hope for in medicine

I am, Sir, yours, &c,, SCEPTICUS.

October, 1863.

To the Editor of the Lancet:

Sir:—In your impression of a late issue appears a letter from Dr. Jones, who states that sixty years ago he saw cases of diphtheria treated by salines, etc. He also says he does not remember having lost a single case.

I, for one, beg most respectfully to doubt the truth of this assertion. In his account of the train of symptoms, he has certainly omitted some of the most prominent ones. His treatment is ridiculous for such a formidable disease. I think nearly all the profession agree that stimulants administered early constitutes the principal part of the treatment. Dr. Jones, moreover, does not name the different preparations containing chlorine. I am sure, after a very great experience in the treatment of this complaint, chlorine in some form or other, both internally and as gargles, is the sheet-anchor in the shape of medicines.

I am, Sir, yours, &c. M D.

October, 1863.

